# Reducing the nanoparticles generated at the wheel–rail contact by applying tap water lubricant at subway train operational velocities

**DOI:** 10.1038/s41598-021-02037-0

**Published:** 2021-12-03

**Authors:** HyunWook Lee

**Affiliations:** Transportation Environmental Research Department, New Transportation Innovative Research Center, Korea Railroad Research Institute, 176 Cheoldo Bangmulgwan-ro, Uiwang-si, 16105 Gyeonggi-do Korea

**Keywords:** Mechanical engineering, Environmental impact

## Abstract

The formation characteristics and the reduction of nanoparticles emitted from wheel–rail contacts at subway-train velocities of 73, 90, and 113 km/h under dry and water-lubricated conditions (using tap water) were studied using a twin-disk rig. The resulting number concentration (NC) of ultrafine and fine particles increased with train velocity under both conditions. Particle generation varied with slip rate under both conditions in both the particle categories. Furthermore, the formation characteristics at 113 km/h under dry conditions showed a notable deviation from those under water-lubricated conditions in three aspects: (i) The maximum NC of ultrafine particles was higher than that of fine particles, (ii) the predominant peak diameter was in the ultrafine particles category, and (iii) the proportion of ultrafine particles was much higher than those of the fine particles. Applying water decreased the NC of ultrafine and fine particles significantly at all tested velocities (by 54–69% and 87–91%, respectively). Adding water increased the NC of particles ≤ 35 nm in diameter, possibly owing to the increase in water vapor and mineral crystals from tap water. Overall, this study provides a reference for researchers aiming to minimize nanoparticle formation at the wheel–rail contacts by applying a lubricant.

## Introduction

The subway system is generally considered a green technology because it uses electricity as power source. However, the underground subway system emits airborne wear particles (AWPs) at various contacts, such as the wheel–rail and brake-pad–disk contacts. These AWPs deteriorate the air quality of the subway environment because they are trapped within the tunnels and easily transported to stations. As many people in metropolitan areas worldwide commonly use subways, concern has arisen regarding the air quality of the subway environment owing to the possible adverse effects of AWPs on public health^1–4^.

Slip at the wheel–rail contact leads to the mechanical wear of the wheel and rail materials and increases the contact temperature. The contact temperatures increase with slip^5,6^ and train velocity^5^. Micro and nanoparticles are mainly produced by mechanical and thermal processes, respectively^7^. AWPs are continuously generated during slip at the wheel–rail contacts^8,9^, which occurs during normal train operations owing to the conical shape of the wheel. AWPs are generated by both the brake system and the wheel–rail contact during mechanical braking^10^; however, the wheel–rail contact is the only source of AWP formation during electrical braking^11^, which is the most commonly used braking method in most subway systems. Therefore, the AWPs formed at wheel–rail contacts are the focus of this study.

AWPs emitted from wheel–rail contacts contain metal elements^2,12,13^, such as Cu, Mn, and Fe, which adversely affect the human health when inhaled^2–4^. Smaller particles are more dangerous, as they are more easily inhaled and penetrate deeper into the pulmonary system. Inhaled ultrafine particles are particularly hazardous, as the bloodstream transports these particles to major organs in the human body^14,15^. Therefore, minimizing the formation of AWPs at wheel–rail contacts is a crucial public health issue.

Research on the formation of AWPs at wheel–rail contacts is still in its infancy. Researchers^8–11,16–18^ have mostly focused on identifying the conditions or parameters most conducive to AWP formation at wheel–rail contacts under dry conditions. Thus, methods for minimizing the formation of AWPs at wheel–rail contacts have rarely been studied. Using a pin-on-disk machine, Abbasi et al.^19^ found that the use of a water-based friction modifier decreased the particle number concentration (PNC) of generated AWPs. Using a twin-disk rig, Lee^20^ showed that the applied load influenced the PNC of generated AWPs when lubricated with water and that the use of a water lubricant decreased the NC of airborne nano and microparticles under different loads. Further, by applying tap water as a lubricant at different train velocities, Lee^21^ found that the total NC of microparticles was reduced to near-negligible values, especially that of nanoparticles was significantly reduced, and that of the total AWPs generated under water-lubricated conditions depended on the train velocity. However, a detailed analysis of the formation characteristics and reduction of ultrafine and fine particles have not yet been performed, although both particle categories are within the category of nanoparticles and are harmful to human health.

The effect of train velocity on the formation characteristics of nanoparticles was examined by Lee under dry^8,18,21^ and water-lubricated conditions^21^. From these studies, Lee found that the generation number of nanoparticles generated increased and the formation characteristics were different at different train velocities under dry and water-lubricated conditions. Furthermore, the NC of nanoparticles generated at train velocities ≤ 45 km/h was not significant^8,18,21^ under dry conditions. However, nanoparticle formation has not yet been examined under either dry or water-lubricated conditions at speeds above 103 km/h, despite the typical subway-train operating speed of 70–120 km/h.

Therefore, as a consecutive study of Ref.^21^, a detailed analysis of the formation characteristics, and the reduction of ultrafine and fine particles emitted at typical operational velocities of subway trains under dry and water-lubricated conditions are performed in this work. The formation of ultrafine and fine particles is examined using a twin-disk rig at train velocities of 73, 90, and 113 km/h under dry and water-lubricated conditions.

## Methods

### Experimental setup, particle sensor, test procedure, and sample disks

**Figure 1:**
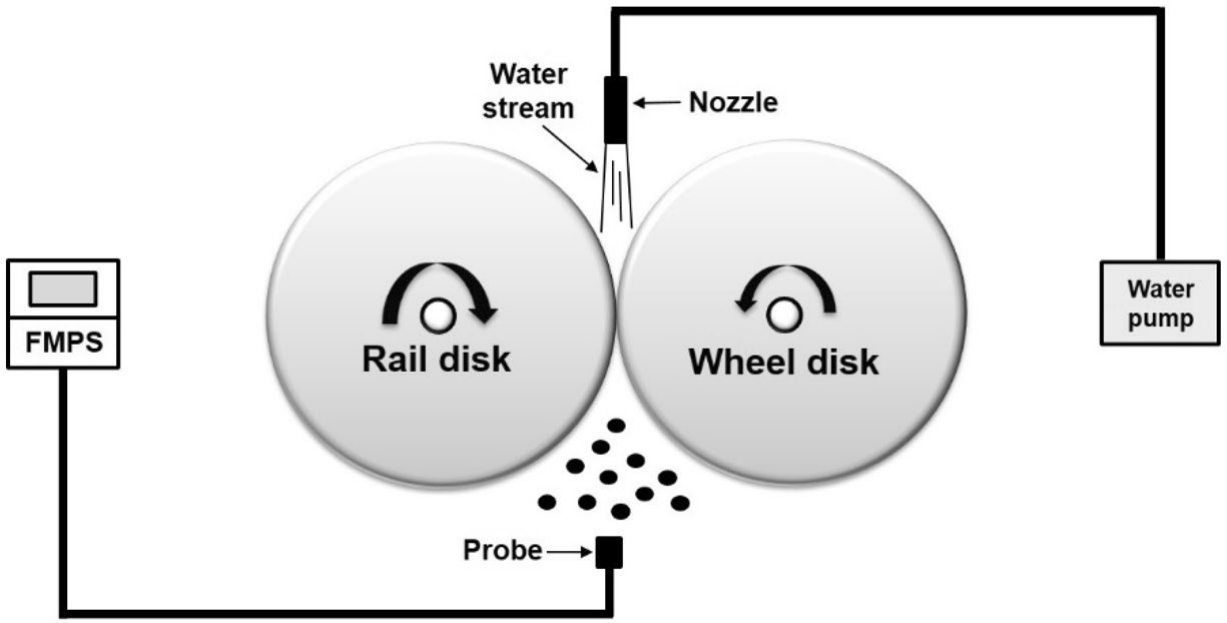
Experimental schematic.

For details regarding the particle sensor and twin-disk rig setup used for simulating AWP formation at wheel–rail contacts, readers can refer to the studies by Lee^20,21^. The scheme of the experiment is shown in Fig. 1. As nanoparticles (*D* ≤ 1 μm, where *D* represents the particle diameter), were the main concern of this study, the NC of ultrafine (*D* ≤ 100 nm) and fine (100 < *D* ≤ 1000 nm) particles formed at the wheel–rail contacts under dry and water-lubricated conditions at different train velocities were measured using a fast mobility particle sizer (FMPS; TSI 3091, USA), which can measure particle diameters ranging from 6 to 560 nm. The nanoparticles were sampled at 1 Hz, the maximum available sampling frequency of the FMPS.

The admixture content and hardness of the employed high carbon steel (SSW–Q1R) disks are summarized in Table 1. The diameter and thickness of the wheel disk were 300 and 135 mm, respectively, and the wheel disk exhibited a flat tread. The rail disk showed a UIC60 head profile, and its diameter and thickness were 300 and 74.3 mm, respectively. The wheel disk showed an initial surface roughness (*R*_*a*_) of 0.24 ± 0.05 μm, whereas the rail disk showed an *R*_*a*_ of 0.26 ± 0.08 μm.

**Table 1:** Admixture content and hardness of the disks.

Disk	Admixture content (wt. %)									Hardness (HB)
	Mn	C	Si	Cr	Cu	Ni	Mo	S	P	
Rail	0.80	0.62	0.25	0.10	0.07	0.04	0.02	0.017	0.016	293
Wheel										285

### Test conditions

Subway trains are typically operated in the velocity range of 70–120 km/h. Thus, to examine the reduction of nanoparticles formed at wheel–rail contacts using water lubrication at the operational velocities of subway trains, tests were performed under dry and water-lubricated conditions at 1300, 1600, and 2000 rpm, corresponding to train velocities of 73, 90, and 113 km/h, respectively. The 15-kN applied normal contact force generated a maximum Hertzian contact pressure of 1200 MPa, which represents the contact between a new wheel and a new rail. A slip rate of 0–3%, which can include pure rolling, rolling/sliding, and pure sliding wheel–rail contact conditions, was employed under dry and water-lubricated conditions. Under water-lubricated conditions, a 7-L/min stream of tap water was applied to the wheel–rail contact point using a water pump. Two trials were performed for each test condition.

### Test procedure

For details regarding the test procedure, readers can refer to the studies by Lee^20,21^. Briefly, the procedure involved the following steps:

1. The background PNCs were measured for 15 s (background range).
2. The rotation rates of the disks were increased for 30 s to the target speeds of 1300, 1600, and 2000 rpm (acceleration range).
3. The 3% slip rate was then realized by maintaining the target speed of the rail disk and linearly increasing the rotation rate of the wheel disk for 120 s (slip range). The slip rate was determined using the following equation:
  1 $${\text{Slip}}\;{\text{rate}}\;(\% ) = \left( {r_{w} {-}r_{r} } \right){/}r_{r} \times 100$$
  where *r*_*w*_ and *r*_*r*_ are the rotation rates of the wheel and rail disks, respectively.
4. The rotation of both disks was decelerated to stop for 45 s (deceleration range).

### Data analysis

To quantify the particle formation at the wheel–rail contacts under dry and water-lubricated conditions, the PNC measured only in the slip range were considered. Under each test condition, the mean PNC measured in the background range was subtracted from that measured in the slip range in each diameter range to consider the effect of the background PNC. The generation characteristics of ultrafine and fine particles and the particle size distribution (PSD) of the nanoparticles measured at each test condition were then examined across the entire slip rate and in the 1% slip rate range. For ease of discussion, slip rate ranges of 0–1%, 1–2%, and 2–3% are referred to as low, moderate, and high-slip zones, respectively.

## Results

**Figure 2:**
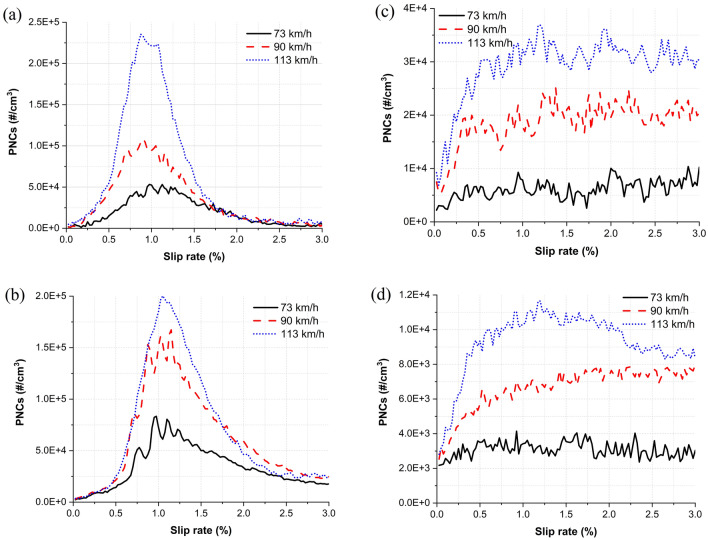
Average total PNCs for all train velocities: (**a**) ultrafine particles (dry), (**b**) fine particles (dry), (**c**) ultrafine particles (water-lubricated), and (**d**) fine particles (water-lubricated).

The average total PNC as a function of the slip rate under dry and water-lubricated conditions are presented in Fig. 2 at the three operational velocities studied. Under dry conditions at each studied velocity, the NCs of ultrafine and fine particles increased with the slip rate up to approximately 1%. Thereafter, they decreased as the slip rate further increased; this transition was more prominent at higher train velocities than at lower train velocities. However, under water lubrication, the NCs in both particle categories increased to a slip rate of approximately 0.5% and stabilized thereafter. Similarly, the increase in the PNC was more prominent at higher train velocities than at lower train velocities.

**Figure 3:**
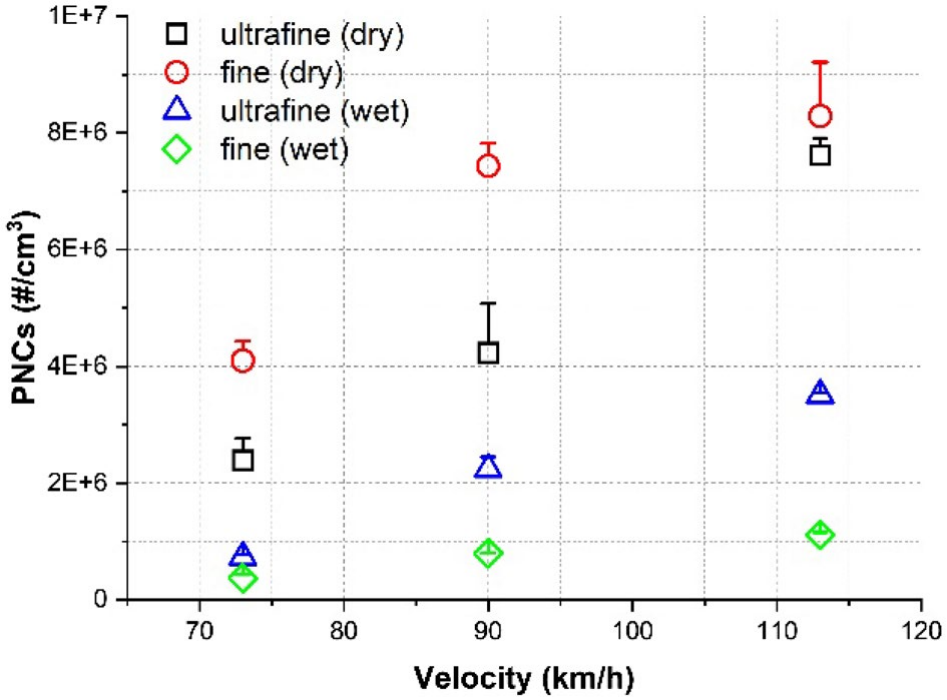
Total NCs of ultrafine and fine particles measured under dry and water-lubricated conditions for each train velocity. Error bars represent one standard deviation.

The dependency of the total NC of ultrafine and fine particles on the train velocity under dry and water-lubricated conditions is presented in Fig. 3. Here, the NC of particles in both particle categories increased nearly linearly with train velocity under both conditions. The total NC of fine particles was higher under dry conditions, whereas that of ultrafine particles was higher under water-lubricated conditions. The total NC of particles in both particle categories was considerably higher under dry conditions.

The average reduction rate of the NC of nano, ultrafine, and fine particles decreased with increasing velocity, as detailed in Table 2. At each velocity, the reduction rate of fine particles was higher than that of ultrafine particles.

**Table 2:** Average NC reduction rates (%) of nano, ultrafine, and fine particles.

Velocity (km/h)	Particle category		
	Nano (%)	Ultrafine (%)	Fine (%)
73	83	69	91
90	74	47	89
113	71	54	87

**Figure 4:**
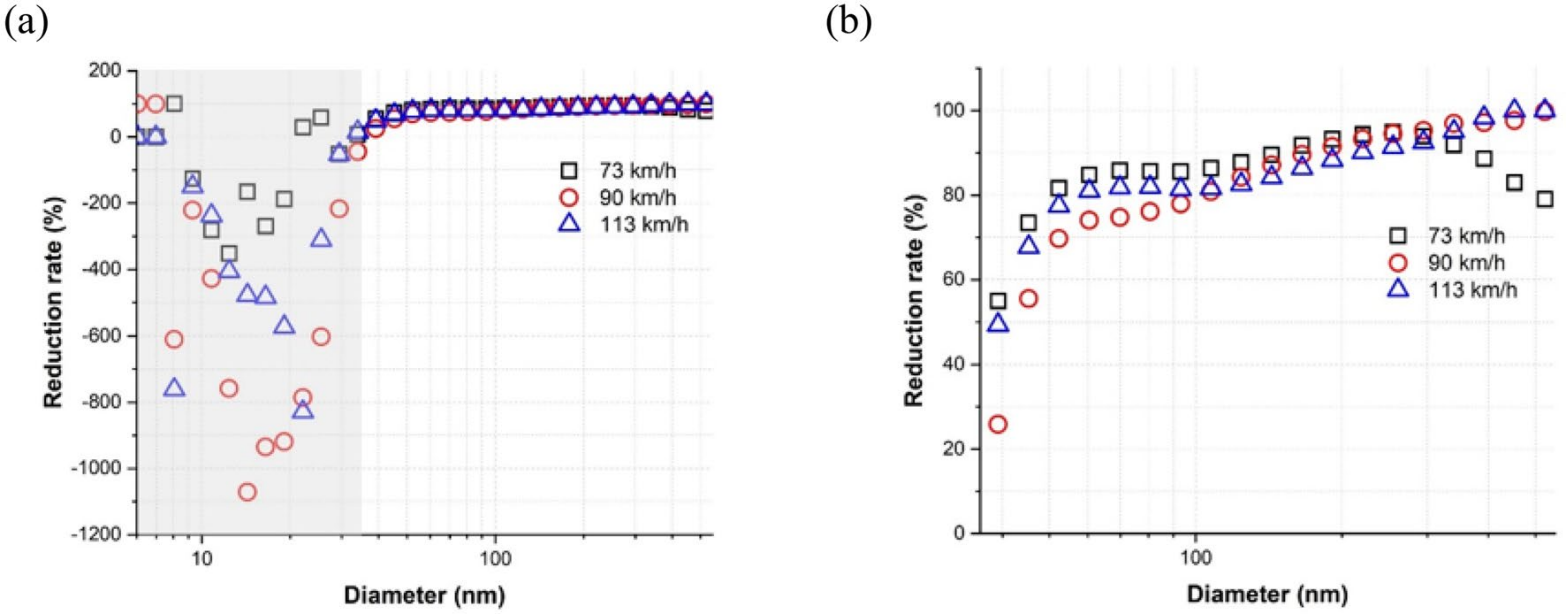
Average reduction rate at each particle diameter at each train velocity: (**a**) entire particle diameters and (**b**) particle diameters ≥ 35 nm. The gray background represents the particle diameter range of approximately 6–35 nm.

At each train velocity, the average NC reduction rate at each particle diameter for all particle diameters measured and for particle diameters larger than approximately 35 nm are presented in Fig. 4a,b, respectively, where the reduction rate at each diameter was determined as

2 $$Reduction\;rate \;(\% ) = \left( {\frac{{1 - PNC_{water{\text{-}}lubricated} }}{{PNC_{dry} }}} \right)_{diameter} \times 100$$

where PNC_water-lubricated_ and PNC_dry_ represent the PNC under water-lubricated and dry conditions, respectively. Thus, a positive value indicates that PNC was lower under water-lubricated conditions. In the particle diameter range of 6–35 nm, represented by the gray background, the reduction rate was negative at more than one train velocity; i.e., more particles of this size were generated under water-lubricated conditions. However, the average reduction rate of particles > 35 nm in diameter was positive for all train velocities studied and increased with increasing diameter, demonstrating a clear reduction of particles by applying a water lubricant. The average reduction rate of particles ≥ 100 nm (i.e., fine particles) was ≥ 80% at each train velocity.

**Figure 5:**
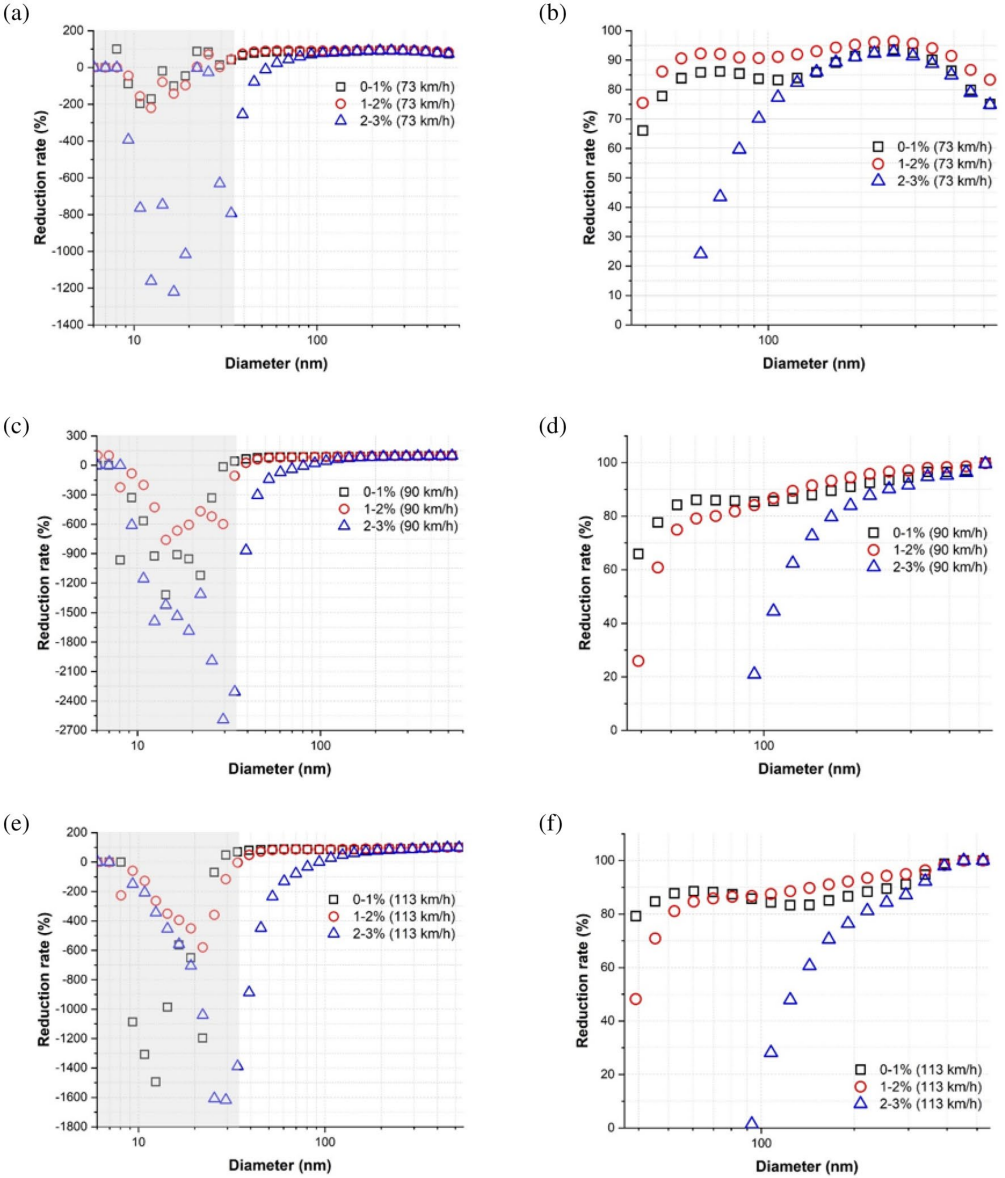
Average NC reduction rate of each particle diameter in each slip rate range studied: (**a**) all particle diameters at 73 km/h, (**b**) particles > 35 nm at 73 km/h, (**c**) all particle diameters at 90 km/h, (**d**) particles > 35 nm at 90 km/h, (**e**) all particle diameters at 113 km/h, and (**f**) particles > 35 nm at 113 km/h. The gray background represents the particle diameter range of approximately 6–35 nm.

The average NC reduction rate of each particle diameter in each slip rate range studied is presented for each train velocity in Fig. 5. The average reduction rate of particles ≤ 35 nm in diameter (the gray region) was negative in the low, moderate, and high-slip zones for all train velocities, indicating that more particles of this size were produced under water-lubricated conditions. However, the average reduction rate of particles > 35 nm was positive in the low and moderate-slip zones; in the high-slip zone, the average reduction rate was positive when the particle size was greater than approximately 60, 90, 90 nm at train velocities of 73, 90, and 113 km/h, respectively.

**Figure 6:**
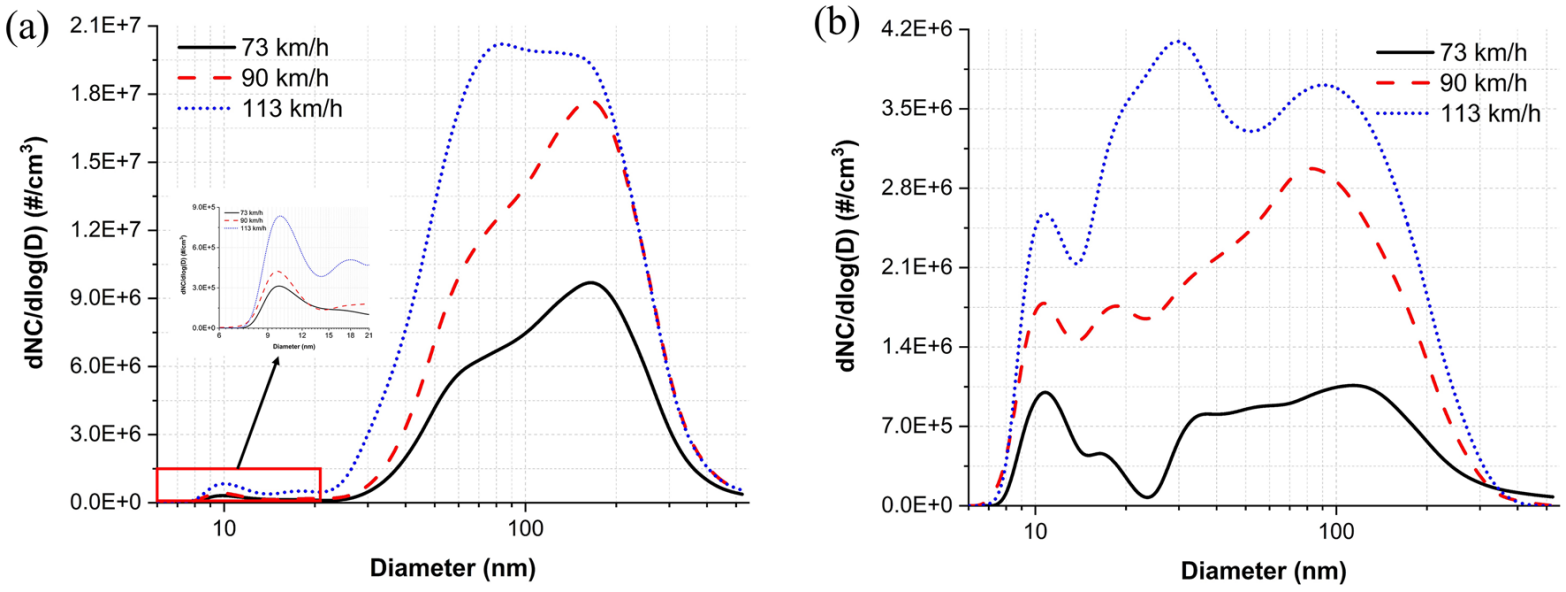
Average PSDs of the nanoparticles measured under (**a**) dry and (**b**) water-lubricated conditions.

The average PSDs of the nanoparticles measured under dry and water-lubricated conditions for the three evaluated train velocities are displayed in Fig. 6. Under dry conditions, the PSDs presented a bimodal shape that peaked at approximately 10 and 170 nm at both 73 and 90 km/h, whereas the PSD at 113 km/h showed a trimodal shape that peaked at approximately 10, 20, and 80 nm. The particle diameter at the predominant peak decreased with train velocity and shifted from the fine (approximately 170 nm at 73 and 90 km/h) to the ultrafine particle category (approximately 80 nm at 113 km/h). The PNC at the predominant peak increased with train velocity.

Under water-lubricated conditions, the PSDs showed a multimodal shape with peaks present at approximately 10, 20, 35, and 120 nm at 73 km/h and a trimodal shape with peaks present at approximately 10, 20, and 80 nm at 90 km/h and 10, 30, and 90 nm at 113 km/h. The particle diameter corresponding to the predominant peak shifted from the fine to the ultrafine particle category, and the diameter decreased with train velocity, i.e., approximately 120, 80, and 30 nm at 73, 90, and 113 km/h, respectively. The PNC at the predominant peak also increased with train velocity. Peak PNC values at approximately 10 nm for each tested train velocity were considerably high under water-lubricated conditions.

**Figure 7:**
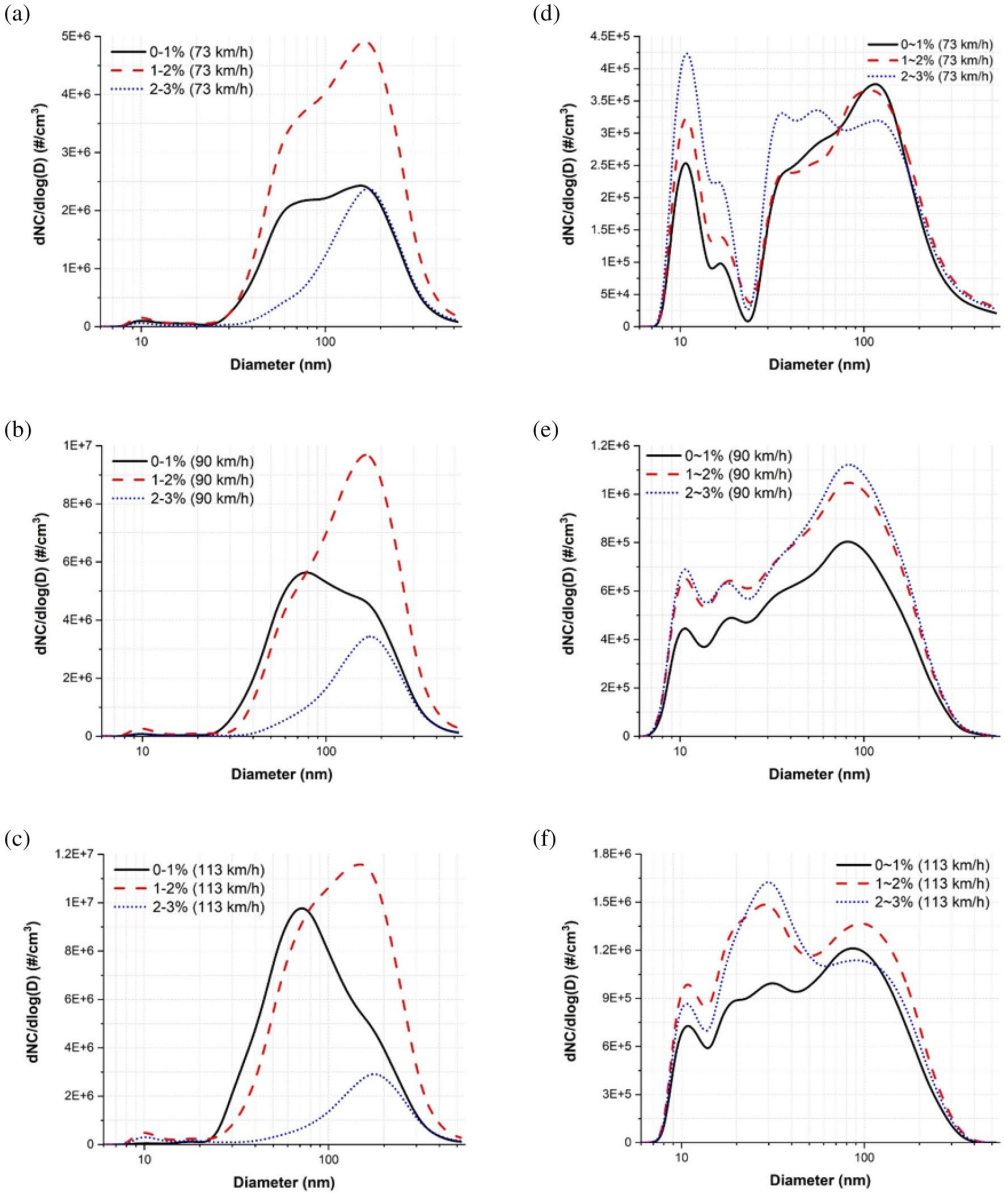
Average PSDs of the nanoparticles measured in each slip rate range under dry conditions at (**a**) 73, (**b**) 90, and (**c**) 113 km/h and under water-lubricated conditions at (**d**) 90, (**e**) 90, and (**f**) 113 km/h.

The average PSDs of the nanoparticles measured at each slip rate range under dry and water-lubricated conditions for the three evaluated train velocities are displayed in Fig. 7. Under dry conditions, the PSDs presented a bimodal shape at 73, and 90 km/h: peaked at approximately 10 and 170 nm at all low, moderate, and high-slip zones at 73 km/h and peaked at approximately 10 and 80 nm at the low-slip zone and at approximately 10 and 170 nm at both moderate and high-slip zones at 90 km/h. The PSDs presented a trimodal shape at 113 km/h that peaked at approximately 10, 20, and 70 nm at the low-slip zone, at approximately 10, 20, and 140 nm at the moderate-slip zone, and at approximately 10, 20, and 170 nm at the high-slip zone. Particle diameters corresponding to predominant peaks remained within the fine particle category at all slip zones at 73 km/h, whereas those shifted from the ultrafine to the fine particle category with increasing the slip zone at 90 and 113 km/h.

Under water-lubricated conditions, the PSDs at 73 km/h showed a trimodal shape that peaked at approximately 10, 20, and 120 nm at the low-slip zone and a multimodal shape that peaked at approximately 10, 20, 35, and 110 nm at the moderate-slip zone and at approximately 10, 20, 35, 50, and 120 nm at the high-slip zone. Those at 90 km/h showed a trimodal shape that peaked at approximately 10, 20, and 80 nm at all slip zones. Those at 113 km/h showed a multimodal shape that peaked at approximately 10, 20, 30, and 80 nm at the low-slip zone and a trimodal shape that peaked at 10, 30, and 90 nm at both moderate and high-slip zones. The particle diameter corresponding to the predominant peak shifted from the fine to the ultrafine particle category with increasing the slip zone, i.e., approximately 120, 110, and 10 nm at the low, moderate, and high-slip zone, respectively, at 73 km/h, whereas those remained within the ultrafine particle category, i.e., approximately 80 nm at all slip zones at 90 km/h and 80, 90, and 30 nm at the low, moderate, and high-slip zone, respectively, at 113 km/h.

## Discussion

Formation characteristics^8,9,18^ and the effect of train velocity on the formation characteristics^8,18^ confirm prior results under dry conditions in both particle categories. At all train velocities, the total NC dependence of ultrafine and fine particles on the slip rate showed an increasing–decreasing trend, with a transition occurring at an approximately 1% slip rate (Fig. 2a,b). The maximum NC of ultrafine and fine particles and the NC at the diameter of the predominant peak increased and the particle diameter at the predominant peak decreased with train velocity (Fig. 6a). The total NCs in both particle categories increased with train velocity (Fig. 3). Fine particles were dominantly formed at all train velocities, although the average proportion of fine particles in the total measured nanoparticles decreased from 63.2 to 52.1% with train velocity.

The contact temperature increases with the slip rate^5,6^ and train velocity^5^. Nanoparticles are mainly produced by thermal processes^7^, and the amount generated increases with the contact temperature^22,23^. Thus, increasing slip rate and train velocity are generally associated with an increase in nanoparticle generation due to the increased contact temperature. Here, the total NC of ultrafine and fine particles increased with slip rate up to 1% and increased faster at higher train velocities. However, the formation of both particles categories began to decrease at all train velocities at a slip rate of approximately 1% (Fig. 2a,b). According to Sundh et al.^24^, a sudden change in the NC of generated nanoparticles indicates a transition in the wear mechanism. The transition to pure sliding from rolling/sliding contact is generally observed at a slip rate between 1 and 2% under dry conditions^25^. This contact transition may cause changes in the wear mechanism and lead to the decrease in particle production seen in this study.

Nanoparticles generation tests have never been performed previously above the train velocity of 103 km/h. The results under dry conditions presented here demonstrate an interesting trend at this velocity up to a slip rate of 1% (Fig. 2a): the maximum NC of ultrafine particles at 113 km/h was much greater than that at 90 km/h. Meanwhile, the NC of ultrafine particles increased sharply at 113 km/h, at a slip rate of 0.5–1%. Additionally, the maximum NC of ultrafine particles was higher than that of fine particles, whereas prior studies conducted by Lee^8,9,18^ reported the opposite trend at train velocities ≤ 102 km/h (i.e., the maximum NC of fine particles was greater than that of ultrafine particles). The predominant peak diameter at 113 km/h was categorized as ultrafine particles, i.e., approximately 80 nm, whereas the peak diameters at 73 and 90 km/h were categorized as fine particles, i.e., approximately 170 nm (Fig. 6a). The proportion of ultrafine particles at 113 km/h was much higher than those at 73 and 90 km/h at the entire slip rate (approximately 36.8%, 36.2%, and 47.9% at 73, 90, and 113 km/h, respectively) and at low slip zone (approximately 45%, 50%, and 63% at 73, 90, and 113 km/h, respectively).

In addition to the increased generation of nanoparticles owing to the increasing contact temperature, the development of thin oxide layers may have amplified the generation of ultrafine particles at 113 km/h, particularly in a slip rate of 0.5–1%. According to Liu et al.^17^, thin oxide layers that generate mostly ultrafine particles can be generated when the contact temperature increases rapidly. Therefore, it is likely that the contact temperature increased rapidly from a slip rate of 0.5–1% at 113 km/h, thus causing the generation of a significant amount of ultrafine particles via thermal processes and the wear of thin oxide layers. The measurement of the contact temperature and particle composition analysis are needed to verify this hypothesis in future studies.

Applying water changed the generation characteristics in both particle categories. At all train velocities, the total NC of ultrafine and fine particles increased up to a slip rate of 0.5%; a near-constant NC was then maintained (Fig. 2c,d). The predominant peak diameters under water-lubricated conditions (approximately 120, 80, and 30 nm for 73, 90, and 113 km/h, respectively) were considerably smaller than those generated under dry conditions (approximately 170, 170, and 80 nm for 73, 90, and 113 km/h, respectively), and they shifted from the category of fine to ultrafine particles as the train velocity increased (Fig. 6b). In addition, the NCs at approximately 10 nm were much higher than those produced under dry conditions, and they increased with train velocity. The measured total NC of ultrafine particles across the entire slip range studied at each train velocity was higher than that of fine particles (Fig. 3); therefore, the proportion of ultrafine particles was higher than 60% at all velocities studied: 66%, 74%, 76% at 73, 90, 113 km/h, respectively.

The train velocity also influenced the generation characteristics of ultrafine and fine particles under water-lubricated conditions, as the total NC of ultrafine and fine particles generated across the entire slip range from wheel–rail contacts increased with train velocity (Fig. 3). The NC at the peak diameters increased with train velocity, and the diameter at the predominant peak was shifted from approximately 120 to 30 nm (Fig. 6b). Ultrafine particles were dominantly generated at all train velocities, and the proportion of ultrafine particles increased from 66 to 76% with train velocity.

Tap water generally contains various minerals such as Na, Mg, Si, and Ca^26^. Small water droplets (≤ 5 μm in diameter) easily evaporate owing to the Kelvin effect^27^. Using a lab-scale nebulizer, Krames et al.^27^ demonstrated that vaporizing sprayed tap water leaves residuals (i.e., mineral crystals) in the air with a PSD peak at approximately 30 nm. When a tap water stream is applied to the contact point, most of the water stream bounces off of the disks’ surfaces as small water droplets. Only a small amount of the water stream applied forms a water film at the contact interface. Further, the high contact temperature causes a portion of the water film to evaporate; evaporated droplets can form water vapor via rapid condensation upon meeting the ambient air. However, the formed residuals remain in the air. Therefore, the observed PNC of particles ≤ 35 nm under water-lubricated conditions may comprise mostly mineral crystals and some water vapor.

The reduction rate of particles approximately 10 nm in size was negative for all train velocities (Fig. 4) and all slip rate ranges (Fig. 5). Further, high NCs of particles of this size were present for all train velocities (Fig. 6b) and all slip rate ranges (Fig. 7d–f). See and Balasubramanian^28^ reported that the highest NC peak occurred at a diameter of approximately 10 nm when boiling water. During typical train operations under dry conditions, contact temperatures can reach hundreds of degrees Celsius^29^, and a flash temperature of up to 900 °C at the asperity level can occur^5^. Although applying water reduces the contact temperature, it likely remains above the boiling point of water. Thus, a portion of the water film evaporates, leading to the formation of water vapor via condensation. The amount of water vapor may increase with increasing train velocity and slip rate due to the higher contact temperature. As the train velocity and slip rate increase, the contact temperature increases. Thus, more water evaporates, leading to the formation of more water vapor. This conclusion was supported by the much higher NC of particles approximately 10 nm in size under water-lubricated conditions than under dry conditions for all train velocities and by the increasing NCs at this diameter with increasing train velocity (Fig. 6b) and slip rate ranges (Fig. 7d–f). Therefore, water vapor is likely the main cause of the peak at approximately 10 nm for all train velocities under water-lubricated conditions.

The increase (i.e., negative reduction rate, Figs. 4 and 5) in particles between approximately 10 and 35 nm in diameter under water-lubricated conditions and with increasing train velocity (Fig. 6b) and slip rate (Fig. 7d–f) was likely owing to the formation of various mineral crystals. An increased train velocity causes water film to exit the wheel–rail contact interface faster, whether as water droplets or via evaporation due to the high contact temperature. Evaporated water film and water droplets can cause mineral crystals to linger in the air. The evaporated water film can also form ultrafine particles by condensing on preexisting nanoparticles in the air. Thus, particles approximately 10–35 nm in diameter could be mostly mineral crystals and ultrafine particles formed via condensation. This phenomenon may increase with train velocity and slip rate, as shown in Figs. 4, 5, 6b, 7d–f.

The reduction rate of ultrafine particles decreased considerably with increasing train velocity from 69% at 73 km/h to 54% at 113 km/h, whereas that of fine particles was consistently high, i.e., above 87%, as summarized in Table 2. As detailed above, more ultrafine particles ≤ 35 nm in diameter were generated with increasing train velocity owing to the generation of water vapor and mineral crystals. Adding a water lubricant increased the NC of particles of this size, thereby drastically reducing the overall reduction rate of ultrafine particles with train velocity.

The increased NCs of ultrafine and fine particles with train velocity can be explained by three main interconnected phenomena. First, the sliding velocity is higher at a higher train velocity at the same slip rate. Thus, a higher contact temperature occurs at a higher train velocity. Second, water film exits the wheel–rail interface faster at higher train velocity, decreasing the thickness of the water film. This may lead to more asperity–asperity contact and/or more contact between oxide layers at the interface, thus increasing the contact temperature. Third, water film in any local area where a high flash temperature occurs evaporates instantaneously, causing locally dry conditions^30^, which then can also lead to more asperity–asperity contact and/or more contact between oxide layers at the interface, thereby leading to a higher contact temperature. For each of these phenomena, increasing the contact temperature causes more water film to evaporate, resulting in more ultrafine and fine particles forming by condensing onto the preexisting nanoparticles. Additionally, increasing the contact between asperities and/or oxide layers can lead to the generation of more metal nanoparticles; in particular, contact between oxide layers generates mostly ultrafine particles^17^. Overall, the combination of these three phenomena may result in increased NCs of ultrafine and fine particles with train velocity under water-lubricated conditions.

Multimodal or trimodal PSDs were seen in Fig. 6, with the highest peak occurring at diameters > 10 nm for all tested velocities, owing to the presence of mineral crystal and metal AWPs formed at the wheel–rail contact. Mineral crystals could be measured when water film and water droplets evaporate, and their sizes are mostly below approximately 40 nm. Additionally, metal vapors and metal AWPs are formed at the wheel–rail contact interface with water vapor even under water-lubricated conditions owing to the contact between asperities and oxide layers. These mineral crystals and metal vapors may also contribute to the PSD peak at approximately 10 nm; further, these particles may have been enlarged by coagulation and condensation, as the same processes occurring onto preexisting mineral crystals and metal particles or water vapors. Thus, these enlarged particles may contribute to the peaks at particle diameters > 10 nm and NCs at particle diameters greater than approximately 35 nm at each velocity under water-lubricated conditions.

Overall, applying water effectively reduced the NCs of both ultrafine and fine particles at all train velocities. In particular, the NCs of fine particles were reduced by 91%, 89%, and 87% at 73, 90, and 113 km/h, respectively. Water addition also reduced the NCs of ultrafine particles by 69%, 47%, and 54% at 73, 90, and 113 km/h, respectively. This reduction in both particle sizes under water-lubricated conditions is likely due to the reduction of contact temperature, which is the main factor causing nanoparticle generation. Applying water can cause boundary lubrication by forming a water film at the contact interface^19^ and causing the development of heavy oxide layers on the wheel and rail surfaces^31^. These two factors can reduce asperity–asperity contacts, resulting in a reduction of the contact temperature. The applied water can also reduce the contact temperature by absorbing the frictional heat and carrying the heat away from the contact interface. Additionally, the water film may trap some of the generated nanoparticles, causing them to be swept away when the water film leaves, rather than becoming airborne^19^.

## Conclusions

In this work, the formation of ultrafine and fine particles from wheel–rail contacts was examined under dry and water-lubricated conditions at the three typical operational velocities of subway trains of 73, 90, and 113 km/h using a twin-disk rig. Tap water was used as a water lubricant. The NCs of ultrafine and fine particles and the PSDs of the nanoparticles measured at each train velocity under dry and water-lubricated conditions were analyzed and compared. The following conclusions were drawn from the obtained results:

1. Inhaled nanoparticles, including those containing metallic elements, are known to have an adverse effect on human health; ultrafine particles may be especially hazardous because they can reach all major organs of the human body via blood circulation. Both ultrafine and fine particles generated from wheel–rail contacts contain such harmful metallic elements. Here, the NCs of both ultrafine and fine particles generated from wheel–rail contacts were significantly reduced by applying water at all tested velocities. Therefore, applying water can help reduce the generation of ultrafine and fine particles that include hazardous metallic elements from wheel–rail contacts at the typical operational velocities of subway trains.
2. The ultrafine and fine particles generated at 113 km/h had different generation characteristics from those generated at 73 and 90 km/h under dry conditions; prior studies have found different generation characteristics at velocities < 50 km/h from at 73–102 km/h in the same test conditions. The generation characteristics of ultrafine and fine particles under dry conditions thus likely vary with the train velocity. Further research is therefore necessary to identify and model the generation of ultrafine and fine particles more accurately over the full range of train velocities, 0–120 km/h.
3. Applying tap water as a lubricant at the wheel–rail contact increased the NC of particles ≤ 35 nm in diameter. This increase was likely caused by an increase in mineral crystals since tap water includes various minerals that can remain as mineral crystals in the air when water droplets and water film evaporate. Future work should aim to confirm this theory by analyzing the chemical compositions of particles of this size generated under water-lubricated conditions.
4. Under water-lubricated conditions, the PSD peak at approximately 10 nm occurred at all tested velocities. Water vapor was assumed to be the main contributor to the high NCs at this particle size; however, mineral crystals and metal vapors may also form in non-negligibly quantities under water-lubricated conditions. Future efforts should aim to clarify the effect of each of these factors.
5. The reduction rate of nanoparticle generation under water lubricated conditions increased with increasing particle diameter at all train velocities. Particles > 35 nm in diameter had positive reduction rates at all train velocities that increased with increasing particle diameter. Further, the reduction rate of fine particles was much higher than that of ultrafine particles; applying a water lubricant reduced the generation of particles ≥ 100 nm in diameter by 80% at all train velocities studied.

## Abbreviations

AWP: Airborne wear particle
NC: Number concentration
PNC: Particle number concentration
FMPS: Fast mobility particle sizer
PSD: Particle size distribution
HEPA: High efficiency particulate air
